# Low-cost chamber design for simultaneous CO_2_ and O_2_ flux measurements between tree stems and the atmosphere

**DOI:** 10.1093/treephys/tpab022

**Published:** 2021-03-02

**Authors:** Juliane Helm, Henrik Hartmann, Martin Göbel, Boaz Hilman, David Herrera Ramírez, Jan Muhr

**Affiliations:** Max-Planck-Institute for Biogeochemistry, Department of Biogeochemical Processes, Hans-Knöll-Str. 10, 07743 Jena, Germany; University of Basel, Department of Environmental Sciences – Botany, Schoenbeinstrasse 6, Basel, Switzerland; Max-Planck-Institute for Biogeochemistry, Department of Biogeochemical Processes, Hans-Knöll-Str. 10, 07743 Jena, Germany; Max-Planck-Institute for Biogeochemistry, Department of Biogeochemical Processes, Hans-Knöll-Str. 10, 07743 Jena, Germany; Max-Planck-Institute for Biogeochemistry, Department of Biogeochemical Processes, Hans-Knöll-Str. 10, 07743 Jena, Germany; Max-Planck-Institute for Biogeochemistry, Department of Biogeochemical Processes, Hans-Knöll-Str. 10, 07743 Jena, Germany; Max-Planck-Institute for Biogeochemistry, Department of Biogeochemical Processes, Hans-Knöll-Str. 10, 07743 Jena, Germany; Georg-August University Göttingen, Department of Bioclimatology, Büsgenweg 2, 37077 Göttingen, Germany

**Keywords:** carbon dioxide consumption, chamber-based measurements, CO_2_ efflux, low-cost sensors, O_2_ influx, oxygen production, respiratory fluxes

## Abstract

Tree stem CO_2_ efflux is an important component of ecosystem carbon fluxes and has been the focus of many studies. While CO_2_ efflux can easily be measured, a growing number of studies have shown that it is not identical with actual *in situ* respiration. Complementing measurements of CO_2_ flux with simultaneous measurements of O_2_ flux provides an additional proxy for respiration, and the combination of both fluxes can potentially help getting closer to actual measures of respiratory fluxes. To date, however, the technical challenge to measure relatively small changes in O_2_ concentration against its high atmospheric background has prevented routine O_2_ measurements in field applications. Here, we present a new and low-cost field-tested device for autonomous real-time and quasi-continuous long-term measurements of stem respiration by combining CO_2_ (NDIR-based) and O_2_ (quenching-based) sensors in a tree stem chamber. Our device operates as a cyclic-closed system and measures changes in both CO_2_ and O_2_ concentration within the chamber over time. The device is battery powered with a >1-week power independence, and data acquisition is conveniently achieved by an internal logger. Results from both field and laboratory tests document that our sensors provide reproducible measurements of CO_2_ and O_2_ exchange fluxes under varying environmental conditions.

## Introduction

Stem CO_2_ efflux is an important part of the carbon balance of forest ecosystems, as it accounts for 5–42% of the total ecosystem respiratory fluxes in forests (Lavigne et al. 1997, Damesin et al. 2002, Chambers et al. 2004, Ryan et al. 2009, Trumbore et al. 2013, Yang et al. 2016). It is typically measured by using chambers of various designs and measurement principles (e.g., Xu et al. 2000, Pumpanen et al. 2004, Maier and Clinton 2006, Saveyn et al. 2008, Etzold et al. 2013, Hilman and Angert 2016, Katayama et al. 2016, Brändle and Kunert 2019) and then often assumed equal, or at least proportional, to the rate of actual respiration in the underlying tissues. This assumption neglects the fact that local CO_2_ emission is the combination of respiratory CO_2_ production and a number of post-respiratory processes (Teskey et al. 2008, Trumbore et al. 2013). Key processes are the transport of dissolved CO_2_ in the xylem both away from or towards the site of measurement (McGuire and Teskey 2004, Teskey and McGuire 2007, Teskey et al. 2008, Bloemen et al. 2013), photosynthetic re-assimilation in chloroplasts of sub-corticular cells (Pfanz et al. 2002, Teskey et al. 2008, Ávila et al. 2014, Cernusak and Cheesman 2015, De Roo et al. 2020), non-photosynthetic refixation by parenchyma cells within the xylem, cambium and phloem via the enzyme phosphoenolpyruvate carboxylase (PEPC) (Gessler et al. 2009, Hilman et al. 2019) or axial diffusion of CO_2_ in the gas phase (De Roo et al. 2019). All these processes can be highly variable over time and may differ between plant organs. Thus, while chambers can provide accurate flux measurements, these fluxes can temporarily differ significantly from local stem respiration rates.

Aerobic respiration not only produces CO_2_ but also results in an anti-correlated uptake of O_2_, as O_2_ is consumed as the electron acceptor at the end of the mitochondrial electron transport chain to form H_2_O. To date, stem O_2_ uptake rates have rarely been measured because the high background of O_2_ in ambient air (20.95 vol.% or 209,500 p.p.m.) makes the detection of O_2_ concentration changes in stem chambers (typically a few hundred p.p.m. over tens of minutes in many chambers) technically challenging. Differential fuel-cell analyzers (e.g., Stephens et al. 2007, Battle et al. 2019) are able to detect very small changes in atmospheric O_2_ (down to several p.p.m.), but require costly infrastructure and high maintenance for application in the field, and usually have a very limited application radius around the position of the analyzer. This can be overcome by laboratory measurements of discrete flask samples from the field, which allow for decentralized measurements over wider areas. Still requiring typically costly analyzers, this approach is usually limited by the number of flasks and the required processing time of the samples and thus typically results in low temporal resolution (Seibt et al. 2004, Hilman et al. 2019). Hilman and Angert (2016) presented an intermediate approach (‘direct discrete method’): they used low-cost chambers that were installed independently of each other on several trees and O_2_ measurements were carried out with a portable optical fiber system. While this approach allows measurements over a wider area and immediate results, this method cannot easily be automated and requires manual measurements, thereby again limiting the temporal and spatial resolution. Cavity-enhanced Raman multi-gas spectrometry (CERS) has been used to measure quasi-continuous fluxes of O_2_ in pine (*Pinus sylvestris* L.) branches (Keiner et al. 2013, 2014, Fischer et al. 2015); however, due to the high sensitivity of the CERS to changes in temperature and air pressure, the methodology is not easily applicable under field conditions.

Like with CO_2_, O_2_ fluxes are affected by processes other than respiration. For example, O_2_ can also be transported to or from the site of respiration by xylem water. However, since O_2_ is ~ 30 times less soluble in water than CO_2_ (Dejours 1981), this effect is considerably smaller. Stem photosynthesis is usually considered to play a minor role in stems of older trees (Wittmann and Pfanz 2008, Rosell et al. 2015, Tarvainen et al. 2018) but would result in a release of O_2_ and an anti-correlated CO_2_ consumption. The only O_2_-exclusive metabolic processes we are aware of is lignification (Amthor 2003) resulting in O_2_ consumption. While the actual amount of O_2_ consumption by lignification is unknown, it seems unlikely to result in large changes of O_2_ concentration in mature trees. Differences between actual respiration and measured fluxes therefore have to be expected for both gases.

The ratio of CO_2_ release to O_2_ uptake (respiratory quotient, RQ) depends on the stoichiometry of the respiratory substrate. For example, the stoichiometric RQ for complete oxidation is ~1 for carbohydrates, ~0.8 for proteins and ~0.7 for lipids. Thus, the measured RQ has been used to identify respiratory substrates (Stiles and Leach 1933, Lambers et al. 2008). Plant respiration is commonly assumed to be dominated by carbohydrate catabolism, but shifts to lower RQ and δ^13^C of respired CO_2_ have been used to infer a switch to lipid respiration for plants under stress and carbon starvation (Tcherkez et al. 2003, Fischer et al. 2015). The simultaneous measurements of CO_2_ and O_2_ fluxes therefore provide a more robust estimate of actual respiration rates as well as information on the stoichiometry of the respired substrate. The much smaller solubility of O_2_ provides the potential to assess the influence of post-respiratory processes on CO_2_ in the stem (Angert and Sherer 2011, Angert et al. 2012, Trumbore et al. 2013, Hilman and Angert 2016, Hilman et al. 2019).

Our aim was to develop and test a portable, weatherproof, low-cost and fully autonomous stem chamber design that allows simultaneous *in situ* measurements of CO_2_ and O_2_ fluxes from tree stems. The data presented here demonstrate the reliability and robustness of the individual sensors as well as the complete chamber design and are based on various laboratory tests and field measurements. Our new tool can improve our understanding of respiratory fluxes in tree stems. Given its low cost, it allows large-scale assessments of ecosystem carbon fluxes with sufficient replication, and the use of O_2_ sensors in addition to CO_2_ sensors represents a substantial improvement for assessing the importance of tree physiological factors in ecosystem carbon fluxes.

## Materials and methods

### Gas sensors

In the final version of our chambers, we measured CO_2_ concentration with the COZIR non-dispersive infrared (NDIR) absorption sensor (Gas Sensing Solution GSS, Cumbernauld, UK), O_2_ concentration with the LuminOx Optical fluorescence quenching sensor (sealed, LOX-02-S; SST Sensing Ltd, Coatbridge, UK) and H_2_O concentration with a high precision humidity sensor (Digital Humidity Sensor SHT-85 (RH/T), Sensirion, ZH, Switzerland). Measurements of H_2_O concentration are necessary for the correction of O_2_ measurements (see 'Correction of measurement data (O_2_) for the dilution effect of changing H_2_O and CO_2_ concentrations'). Initially, we instead used the relative humidity sensor integrated in the COZIR. However, detailed laboratory tests revealed significant mismatches between known and measured humidity and a very slow reaction time for the COZIR built-in sensor. Therefore, we switched to the more accurate and rapidly responding SHT-85 sensor, using the manufacturer’s calibration (detailed humidity test results from the laboratory and field can be found in S1 and S2 available as Supplementary data at *Tree Physiology* Online).

The COZIR has a CO_2_ measurement frequency of 2 Hz and includes a temperature (°C) and relative humidity (%) sensor. Using the internal filter feature, we set the sensor to report running means of the last 50 measurements or 25 s. We do this to reduce high frequency noise and smooth the CO_2_ readings, though this leads to an overall slower response time to concentration changes (details see GSS Sensor User’s Manual 2015). The COZIR sensor allows for one-point calibration (see `Multiple sensor calibration and testing unit').

The LuminOx O_2_ sensor measures the partial pressure of O_2_ (ppO_2_; (mbar)), the total pressure (pO_2_; (mbar)) and temperature (TO_2_; (C^o^)). The O_2_ sensors do not allow for changing the manufacturer-supplied calibration parameters unique to each sensor, which are determined by exposing the sensor to different oxygen concentrations, temperatures and barometric pressures in an environmental chamber. Device specifications are provided in the datasheet of the supplier (see also Table 1).

**Table 1:** Specifications of COZIR-AH-1 and LuminOx sealed optical oxygen sensor

	COZIR ambient sensor AH-1	LuminOx sealed optical oxygen sensor
Mechanism principle	Non-dispersive infrared (NDIR)	Fluorescence quenching
Accuracy/resolution	±0.005% (= 50 p.p.m. ± 3%)	0.1%
Operating temperature	0–50°C	−30–60°C
Relative humidity	0–95%	0–99%
Measurement range	0–10,000 p.p.m.	0–25%
Sensor output	CO_2_ (p.p.m.) Temperature (°C) Relative humidity (%)	ppO_2_ O_2_ (%) Barometric pressure (mbar) Temperature (°C)
Manuals	http://www.co2meters.com/Documentation/Manuals/Manual-GSS-Sensors.pdf	https://www.sstsensing.com/wp-content/uploads/2017/07/DS0030rev13_LuminOx.pdf

The SHT-85 humidity sensor allows measurements in the range of 0–100% RH with an accuracy of ±1.5% RH (Sensirion Datasheet, Digital Humidity Sensor SHT-85 (RH/T) 2018).

### Chamber design and measurement principle

**Figure 1.**
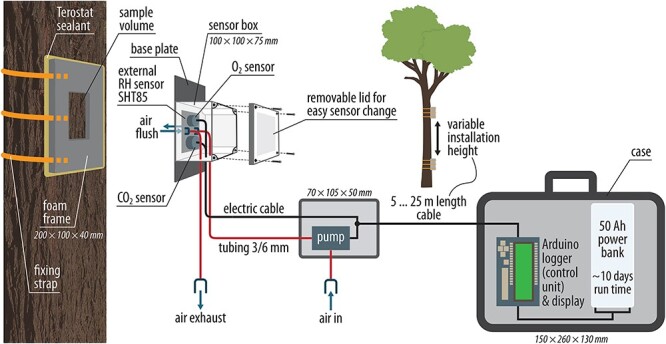
Custom-made modular stem gas exchange system featuring a CO_2_ and an O_2_ sensor for repeated cyclic measurements of changes in gas concentration over time within a closed dynamic stem chamber installed on the tree stem. A pump automatically flushes the chamber headspace in between measurement cycles. The device is battery powered with up to 10 days operating time. An Arduino® controls switching between measurement and flushing mode and logs the data on an SD card. ^©^Annett Börner

We designed a modular measurement system (Figure 1; see S3 available as Supplementary data at *Tree Physiology* Online) consisting of (i) the chamber module for creating a gas tight measurement headspace on the stem surface; (ii) a waterproof housing for the CO_2_ and O_2_ sensors mounted on top of the chamber; (iii) a separate housing for a pump; and (iv) a waterproof transport-case containing the power supply and an Arduino® logging and control unit. Our device operates as a closed-cycle system, alternating between incubation periods for measuring CO_2_/O_2_/H_2_O concentration changes over time, and periods for flushing the chamber headspace with ambient air.

The chamber module is made of a 5-mm-thick polyethylene high-density sheeting and is 10 cm wide and 20 cm long, mounted on a 4-cm-thick closed-porous cell foam (EPDM, ethylene propylene diene monomer rubber). The foam is placed between the plate and the tree stem prior to fixing the chamber module with three ratchet straps. If required for an air-tight fit, the bark underneath the foam can be smoothed with sandpaper or an angle grinder, but this requires extreme caution to avoid any damage to the underlying cambium and phloem. Putty butyl sealant (Teroson RB IX, Henkel, Düsseldorf, Germany) is then applied around the edges of the foam to cover potential small leaks associated with remaining bark irregularities. Installed chambers are tested for leaks by blowing high CO_2_ air around the edges while monitoring the headspace CO_2_ concentration. Leaks are closed with putty and by refastening the straps until repeated tests show no further leaks. Because chamber headspace volume varies with stem geometry and ratchet strap tension from ca 75 to 112 cm^3^, it is measured for each installation by filling the headspace (*in situ*) with water from a calibrated syringe. To do so, we use two syringe needles (inserted from the top of the chamber, between bark and foam, to reach the headspace volume), one for injecting the water, the other to vent air from the chamber until water droplets appear. Sensors should be removed during headspace measurement.

The CO_2_, O_2_ and H_2_O sensors are placed inside a waterproof (IP66 standard) housing (acrylonitrile-butadiene-styrene copolymers; dimensions: 10 cm L × 10 cm W × 7.5 cm H) with a removable lid. For easier handling, the sensor box is permanently welded to the base plate (polyethylene high-density) of the chamber module. The sensors are placed over drill holes on 1-cm-thick EPDM rubber rings, allowing gas diffusion from the chamber headspace into the sensors. The O_2_ sensor has a sealed sensor base (LOX-02-S) to guarantee that gas from the chamber headspace cannot leak through the sensor into the ambient air. For the CO_2_ sensor, such a design is currently not available, so we seal the sensor base by applying hot glue around the electronic pins. The polyethylene plate and the sensor box are fully covered with adhesive aluminium foil to prevent heating from sun exposure. We further reduce potential temperature variations by installing the chambers on the side of the tree receiving the least direct sunlight during the day (i.e., north in the northern hemisphere). The air inlet and outlet of the chamber are connected to the pump via metric tubing (PVC tubing, RS Components GmbH, Frankfurt am Main, Germany) with a diameter of 6 mm (outer) x 3 mm (inner). The sensors (CO_2_, O_2_ and H_2_O) are connected to a 5 V power bank (52,800 mAh, Li-ion type, MP-50000, XTPower, Seattle, WA, USA) in a separate waterproof transport-case (72,601 Outdoor Dry Box, Dyntronic GmbH, Glashütten, Germany), allowing the system to remain operational unattended for up to 10 consecutive days. The chamber operation and data logging are controlled by a custom-made Arduino® (Arduino Mega 2,560 Rev3, Arduino S.r.L.) device (see S4 available as Supplementary data at *Tree Physiology* Online). The Arduino® processor-based device enables us to program the measurement time interval, incubation time, duration of chamber headspace flushing and to define a CO_2_ concentration threshold in which chamber flushing is desired. Our measurement approach relies on the ability to accurately measure concentration change rather than absolute concentrations; hence, we focus here on the rates of change of both gases over time. For a component list of the stem chamber (main parts), see S5 available as Supplementary data at *Tree Physiology* Online.

### Multiple sensor calibration and testing unit

We built a multiple sensor calibration and testing unit, consisting of a gas-tight chamber with a volume of 1945 ml featuring two valves for flushing and a series of slots for simultaneous operation of up to 10 LuminOx and 10 COZIR sensors (see S3 available as Supplementary data at *Tree Physiology* Online). All 20 sensors are connected to a computer via multiple USB hubs.

For calibration of the COZIR sensors, we used one reference gas of known CO_2_ concentration (approx. half of the maximum sensor range, here referred to as ‘span’ gas). First, we flushed the calibration unit for 10 min at 2 l min^−1^ with span gas, then closed it and let the reading stabilize (SD ≤ 30 p.p.m. for CO_2_ for at least 10 measurements) before the respective calibration parameter was adjusted according to the span gas concentration (program: Microsoft Visual Basic). The calibration program allows reading and logging of data, provides access to the filter setting of the COZIRs and for defining stabilization criteria, and (when readings stabilize) sensors can be calibrated to known gas standard. The COZIR sensors allow to automatically store the new calibration parameters internally.

Direct calibration of the LuminOx sensors was not possible since there is no option to adjust their internal calibration parameters. We therefore conducted indirect calibration by comparing the sensors' measurement to known O_2_ concentrations of gas mixtures being measured. The focus was on accurately measuring changes of the O_2_ concentration rather than the absolute concentration since concentration changes over time are the parameter the chamber flux measurements are based upon. We placed the LuminOx sensors in the calibration unit and exposed them to synthetic air with an O_2_ concentration of approx. 20.95% O_2_. We diluted the headspace concentration by injection of 30 ml pure N_2_ into one port of the unit while extracting 30 ml of the unit’s air on the opposite side to keep pressure in the chamber constant. We recorded the measurement after equilibration (i.e., SD ≤0.01% for at least 10 measurements) and repeated the dilution several times.

### Testing of the sensors under laboratory and field conditions

We performed the following tests to validate accuracy and linearity of the COZIR and LuminOx sensors: measurements of a range of known concentrations (CO_2_, O_2_) under (i) standard laboratory conditions; (ii) under varying temperatures; (iii) at increasing time intervals since last calibration (sensor drift); (iv) measuring the headspace over germinating wheat seeds as a biological model system of carbohydrate catabolism (with an expected ratio of CO_2_ production: O_2_ consumption of 1); (v) direct comparison of the COZIR sensor with another widely used commercially available CO_2_ sensor (GMP252, Vaisala GmbH, Helsinki, Finland) under field conditions; and finally (vi) a field application test of the complete chamber setup.

#### Measurements of known gas concentrations under standard laboratory conditions

As a reference point, sensor readings were tested against a range of known concentrations. Sensors were mounted in the calibration and testing unit, which was subsequently flushed with known gas concentrations. For the COZIR sensor, we used calibrated reference gas bottles (Westfalen AG, Münster, Germany) with CO_2_ concentrations of 420, 2944 or 6020 p.p.m.. For O_2_, we followed the same procedure as described in the `Multiple sensor calibration and testing unit'. We performed three dilution steps (20.95, 20.68, 20.41 and 20.16% representing the mean O_2_ concentration from 20 LuminOx sensors at 25°C).

#### Measurements of known gas concentrations: effect of temperature changes and sensor drift over time

For extended field application, it is important to test whether temperature changes or sensor drift over time affect the gas measurements. All tests were performed using 10 CO_2_ and/or 10 O_2_ sensors. For the temperature test, the equipment (including the reference gas bottles) was set up in a phytochamber where we could control ambient temperature. Tests for the COZIR and LuminOx sensors were done separately. Tests started by adjusting the phytochamber temperature to either 5, 10, 20 or 25°C (for COZIR sensors) or 5, 15 or 25°C (for LuminOx sensors), followed by a period for equilibration of all materials and gas cylinders. We then followed the same procedure as explained in the `Measurements of known gas concentrations under standard laboratory condition' (repeated for the different temperature levels).

Sensor drift was determined by repeatedly testing field installed sensors over a period of 3 weeks without re-calibration, each time measuring known gas concentrations (CO_2_: 420, 1430, 2944 and 6020 p.p.m.; O_2_: 20.95, 20.68 and 20.41%). Sensor readings were tested before initial installation see “Measurements of known gas concentrations under standard laboratory conditions”. The sensors used in this drift test were installed and recorded data in the field between successive tests. They were tested in the lab 14, 18 and 22 days after initial installation and then re-installed in the field without re-calibration.

#### Incubation of germinating wheat seeds

We tested sensor performance by measuring the CO_2_ emission and O_2_ uptake of germinating wheat seeds. While absolute fluxes in this setup are unknown, the ratio of CO_2_ production to O_2_ consumption (respiratory quotient, RQ) is expected to be 1 since wheat seeds exclusively use carbohydrates as respiratory substrates (Stiles and Leach 1933, Lambers et al. 2008). Wheat seeds were soaked in water overnight and placed in the calibration and testing unit (laboratory conditions, 25°C). Headspace concentrations were measured for 80 min (*n* = 10 for COZIR and LuminOx sensors, respectively). Corrections for the dilution effect on O_2_ (by CO_2_ and H_2_O) were implemented (see `Correction of measurement data (O_2_) for the dilution effect of changing H_2_O and CO_2_ concentrations').

#### Sensor comparison under field conditions: COZIR vs Vaisala GMP252

COZIR measurements were compared to the Vaisala GMP252 NDIR CO_2_ sensor frequently used for xylem CO_2_ measurements inside the stem (e.g., Saveyn et al. 2008, Cerasoli et al. 2009, Bloemen et al. 2014, Salomón, Valbuena-Carabaña, Teskey, et al. 2016, Fan et al. 2017). The Vaisala GMP252 has a measurement range of 0–10,000 p.p.m. CO_2_ with accurate p.p.m.-level CO_2_ measurements (device specifications are provided in the datasheet of the supplier: Vaisala GMP252 Carbon Dioxide Probe 2020). We designed a special version of our chamber system for simultaneous measurements of both the COZIR and the Vaisala GMP252 sensor in the same chamber under field conditions. Three such chambers were installed at three positions on the same tree (*Prunus avium* L., mean stem diameter: 105 cm) and measured CO_2_ efflux for 1 week in September 2019 (14th–20th) in Jena, Thuringia, Germany.

**Figure 2.**
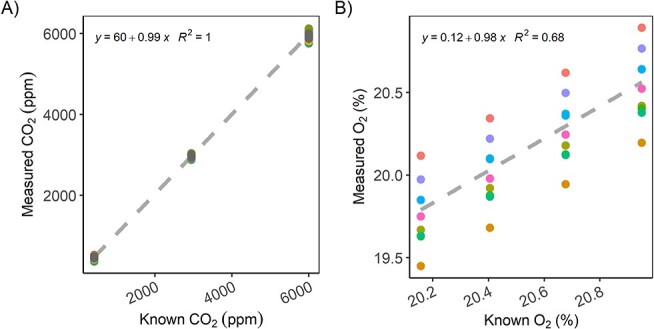
Measurements of known gas concentrations in the calibration and testing unit (25°C) for (A) CO_2_ (CO_2_ concentrations of 420, 2944 or 6020 p.p.m.) by COZIR sensors (*n* = 10) and (B) O_2_ (in %, dilution from 20.95 to 20.16%) by LuminOx sensors (*n* = 10). Colours depict different sensor ID. The dashed line represents the linear regression.

#### Field application test

Parallel to our sensor test in the laboratory, we tested several chambers under field conditions. These field tests (see `CO_2_ and O_2_ flux measurements in the field') were conducted in the Thuringian Forest; Germany (Oberschönau, 50°71'N, 10°6′E) at three mature poplar trees at 1.3 m stem height during July 2019 (stem diameter: 95.5–131.5 cm; ~70 year old). During this stage, relative humidity was still measured with the integrated COZIR RH sensor that later was replaced by the more accurate SHT-85. Corrections for the dilution effect on O_2_ (by CO_2_ and H_2_O) were implemented (see `Correction of measurement data (O_2_) for the dilution effect of changing H_2_O and CO_2_ concentrations').

### Correction of measurement data (O_2_) for the dilution effect of changing H_2_O and CO_2_ concentrations

Oxygen as a non-trace gas is sensitive to concentration changes of any other gas (Keeling et al. 1998). Measured apparent O_2_ concentrations thus have to be corrected for changes in CO_2_, H_2_O and even for changes in O_2_ itself (self-dilution) (Keeling et al. 1998, Bugbee and Blonquist 2006). We would like to clarify that these corrections have nothing to do with a cross-interference of other gases on the sensor signal, and we are not aware of any cross-interference issues in the sensors we used.

Previous publications usually linked the correction with simultaneously expressing the O_2_ concentration changes relative to an arbitrarily defined reference concentration in per meg (= per million), thus following the original method as described by Keeling et al. (1998). This conversion undeniably has clear advantages when aiming at reporting and comparing measurements of absolute atmospheric O_2_ concentrations and their changes over time. However, considering the focus and target group of our new application, we decided to go for a more intuitive approach that does not require the reader to get acquainted with the per meg scale. Since we are interested in simultaneous concentration changes of CO_2_, O_2_ and H_2_O during separate incubation cycles, we refrained from expressing all concentrations relative to a fixed standard, and rather decided to express all concentrations as relative to the starting concentration ([CO_2_]_*t* = 0_, [O_2_]_*t* = 0_, [H_2_O]_*t* = 0_) during a measurement cycle. The measured apparent change in the O_2_ concentration (δO_2,app_) at any given time during a measurement cycle is correcting for the effect of any observed changes in the concentration of CO_2_ and H_2_O relative to the starting concentration (δCO_2_ and δH_2_O). These corrections are proportional to the mole fraction of O_2_ in the gas mixture, i.e., X_O2,*t* = 0_, which equals the apparent O_2_ concentration in p.p.m. divided by 10^6^.

\begin{eqnarray*} \delta{\mathrm{O}}_{2,\mathrm{corr}}\left[\mathrm{p.p.m.}\right]=\frac{\delta{\mathrm{O}}_{2,\mathrm{app}}\left[\mathrm{p.p.m.}\right]+\delta{\mathrm{CO}}_2\left[\mathrm{p.p.m.}\right]\times{{\mathrm{X}}_{{\mathrm{O}}_2}}_{t=0}+\delta{\mathrm{H}}_2\mathrm{O}\left[\mathrm{p.p.m.}\right]\times{{\mathrm{X}}_{{\mathrm{O}}_2}}_{t=0}}{1-{{\mathrm{X}}_{{\mathrm{O}}_2}}_{t=0}}. \ (1) \end{eqnarray*}

For a description of the original approach described by Keeling et al. (1998) (extended by a H_2_0 correction), see S6 available as Supplementary data at *Tree Physiology* Online. Please note that the sensors we used do not directly measure the concentration of water in p.p.m., they rather measure the relative humidity. To convert relative humidity into an absolute H_2_O concentration, we first calculated the saturation water vapor pressure (es, hPa) as a function of the chamber temperature T (°C) with the Clausius–Clapeyron relation (Bugbee and Blonquist 2006)

(2) \begin{equation*} \mathrm{es}\ \left(\mathrm{hPa}\right)=6.11\ \mathrm{\times}\ \exp \left(\frac{(17.502 \mathrm{\times} T)}{(T + 240.97)}\right) \end{equation*}

Since relative humidity is 100% at saturation vapour pressure, the ambient partial pressure of H_2_O (ea, hPa) can be calculated as a function of the current relative humidity

(3) \begin{equation*} \mathrm{ea}=\mathrm{es}\ \mathrm{\times}\ \mathrm{RH}. \end{equation*}

The current concentration of H_2_O thus equals the ratio of ea to the total atmospheric pressure of all air inside the chamber (P), which we report in p.p.m. here

(4) \begin{equation*} {\mathrm{H}}_2\mathrm{O}\ \left(\mathrm{p.p.m.}\right)=\frac{\mathrm{ea}}{P\ \mathrm{\times}\ 1{0}^6}. \end{equation*}

### Flux calculation and apparent respiratory quotient

The CO_2_ and O_2_ fluxes (*F*, μmol m^−2^ s^−1^) were calculated according to the following equation:

(5) \begin{equation*} F=\frac{\Delta C}{\Delta t}\ \mathrm{\times}\ \frac{V}{A\ }\ \mathrm{\times}\frac{P}{R\ \mathrm{\times}\ T}, \end{equation*}

where Δ*C*/Δ*t* (hereafter referred to as slope) is the change in concentration of gas C (in p.p.m.) over time *t* (s^−1^) for CO_2_ and O_2_, respectively. *V* is the volume of the chamber (m^3^), *P* the barometric pressure (kPa), *R* the molar gas constant (0.008314 m^3^ kPa K^−1^ mol^−1^), *T* the temperature (K) and *A* the stem surface area (0.0028 m^2^). *P* and *T* are recorded from the sensors. We assumed linearity in the first 20 min of measurement (time is required to measure changes in the O_2_ and CO_2_ concentration of at least 1000 p.p.m., 0.1%) and therefore used the slope of the linear regression to calculate CO_2_ (increasing concentration) and O_2_ (decreasing concentration) change over time. The negative slope of O_2_ is always given as absolute value. Only measurements with correlation coefficient (*R*^2^) > 0.96 for both O_2_ and CO_2_ were used. Lower correlation coefficients were discarded. For field data, the first 5 min of each measurement cycle (user-defined) were discarded after we noticed these data are noisy, probably due to pressure fluctuations after the pumping period. The sensors’ readings were extracted every 10 s.

The ratio of CO_2_ efflux and O_2_ influx results in the Apparent Respiratory Quotient (Angert and Sherer 2011; ARQ; Eq. (6)) as we do not measure the actual RQ of respiring cells (see `Incubation of germinating wheat seeds') but the stem equivalent of RQ

(6) \begin{equation*} \mathrm{ARQ}=\frac{\mathrm{C}{\mathrm{O}}_2\ \mathrm{production}}{{\mathrm{O}}_2\ \mathrm{consumption}}. \end{equation*}

### Statistical analysis

Data from temperature and drift tests were analyzed by regression analysis. As shown in Eq. (5), flux rates for the chambers were calculated from concentration changes over time. Thus, sensors have to reliably measure concentration changes. We estimated how much the slope of a linear regression of measured concentration vs known concentration was affected by temperature, or time (drift). We examined the slope of the regression, where slope of 1 represents a perfect agreement, slope <1 means the sensors underestimating the true concentration change and vice versa. Slopes measured by individual sensors (*n* = 10) were tested for temperature and time effects. For the wheat seed respiration test, linear regressions and comparisons among slopes of CO_2_ increase and O_2_ decrease over time were performed, too. For normally distributed data (after checking the assumption of heteroscedasticity with a Levene test) one-way ANOVA was used for comparison among slopes. Correlation between COZIR and Vaisala GMP252 was evaluated by Pearson’s correlation coefficient. Data from CO_2_ and O_2_ flux measurements in the field (see `CO_2_ and O_2_ flux measurements in the field') were combined to 4 h mean for further analysis. All statistical analyses were conducted in R 3.4.4 (RStudio Team 2016).

## Results

### Measurements of known gas concentrations

Measurements of known CO_2_ concentrations by the COZIR sensors showed good accuracy and precision (Figure 2A). Using 10 sensors, the linear regression for measured vs known concentration had a slope of almost unity (0.99) and variability between the individual sensors was very small as reflected in the *R*^2^ of 1.0.

For the LuminOx sensors (Figure 2B), measurements of known O_2_ concentrations also resulted in a slope for the linear regression close to 1 (0.98), indicating that the sensors can reliably detect relative concentration changes over the tested range. However, the variability between individual sensors in terms of absolute concentration measurements was far greater than for the COZIR sensors, with individual sensors being off by almost ±0.5%. This results in a low *R*^2^ of the regression of only 0.68.

### Measurements of known gas concentrations as affected by temperature changes, and sensor drift over time

Changes in temperature did affect O_2_ but not CO_2_ measurements. For CO_2_ measurements, linear regression parameters of measured vs known concentrations show no statistically significant effect of temperature with mean slopes (±SD) of 0.96 ± 0.05, 0.97 ± 0.03, 0.99 ± 0.01 and 0.97 ± 0.02 at 5, 10, 20 and 25°C, respectively (Table 2). For O_2_, differences in temperature were highly significant with mean slopes (±SD) of 0.81 ± 0.06, 0.86 ± 0.03 and 0.97 ± 0.02 at 5, 15 and 25°C, respectively (Table 2). The sensors themselves do not differ significantly (ANOVA, COZIR: *F* = 0.12, *P* = 0.73; LuminOx: *F* = 0.43, *P* = 0.52). Based on these findings, we formulate the following temperature correction for the O_2_ sensors:

**Table 2:** Rate of change (slope) for different temperature levels and sensor drift over time (mean ± SD). Analysis of variances (ANOVA) for temperature and drift effect on COZIR and LuminOx reading (*n* = 10) is shown. Significant results are shown in bold.

		COZIR-AH1		LuminOx	
Effect (temperature/duration)		mean slope ± SD	ANOVA (*F*, *P*)	mean slope ± SD	ANOVA (*F*, *P*)
Temperature (°C)	5	0.96 ± 0.05	*F* = 0.21, 0.65	0.81 ± 0.06	***F* = 80.31, <0.001**
	10	0.97 ± 0.03		n.a.	
	15	n.a.		0.86 ± 0.03	
	20	0.99 ± 0.01		n.a.	
	25	0.97 ± 0.02		0.97 ± 0.02	
Drift (*d*)	14	0.97 ± 0.05	*F* = 2.78, 0.055	1.02 ± 0.10	*F* = 0.10, 0.96
	18	0.96 ± 0.05		1.03 ± 0.10	
	22	0.95 ± 0.04		1.03 ± 0.08

(7) \begin{equation*} \mathrm{SC}({\mathrm{O}}_2)=-0.010\ \mathrm{\times}\ T\ (\mathrm{in} \ {}^{\circ}\mathrm{C})+1.30 \end{equation*}

where SC is the slope correction factor that should be multiplied with the measured slope in stem chamber incubation in temperature *T*. As fluxes were estimated based on slope changes over time (see `Flux calculation and apparent respiratory quotient'), only a slope correction was considered.

Time elapsed since last calibration had an effect on CO_2_ measurements when exceeding 22 days, i.e., sensor drift affected the CO_2_ sensors after ca 3 weeks (Table 2). Shorter time intervals (14 and 18 days) showed no significant effect on CO_2_ measurements. Mean slopes (±SD) from linear regression parameters of measured vs known CO_2_ concentrations were 0.97 ± 0.05, 0.96 ± 0.05 and 0.95 ± 0.04 after 14, 18 and 22 days of field operation, respectively. We found no effects of sensor drift over time for the O_2_ sensors, with mean slopes (±SD) of 1.02 ± 0.10, 1.03 ± 0.10 and 1.03 ± 0.08 after 14, 18 and 22 days of field operation, respectively. The sensors themselves do not differ significantly (ANOVA, COZIR: *F* = 1.06, *P* = 0.44; LuminOx: *F* = 1.36, *P* = 0.28).

### Incubation of germinating wheat seeds

**Figure 3.**
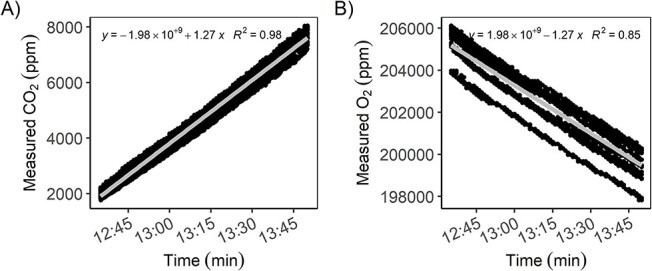
Wheat seed respiration within the calibration and testing unit (25–27°C) over 80 min. (A) Increase of CO_2_ (p.p.m.) over time and (B) decrease of O_2_ (p.p.m.) is shown (*n* = 10 for CO_2_ and O_2_, respectively). Linear regression was used to determine the rate of change.

Over the 80-min incubation period of germinating wheat seeds, the 10 CO_2_ sensors reported a mean (±SD) increase of the CO_2_ concentration of 5625 ± 302 p.p.m., while the 10 O_2_ sensors reported a mean decrease of the O_2_ concentration of 5800 ± 300 p.p.m., i.e., concentration changes over time were anti-correlated and not significantly different with regard to absolute changes (CO_2_: slope = 1.27 ± 0.02, *R*^2^ = 0.98; O_2_: slope = −1.27 ± 0.05, *R*^2^ = 0.85, Figure 3); resulting in an RQ value (±SD) of 1.00 ± 0.03.

### Comparison of CO_2_ measurements between COZIR and Vaisala sensors

**Figure 4.**
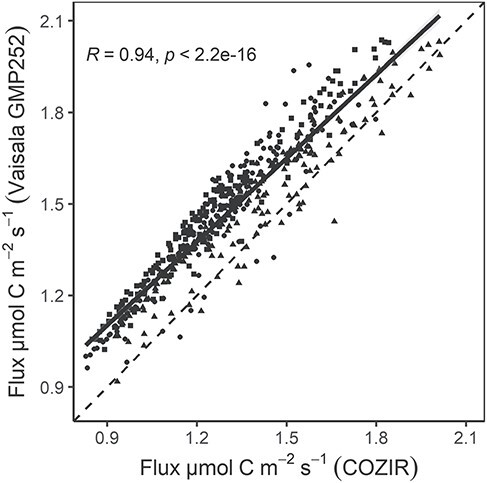
Scatter plot showing the calculated CO_2_ fluxes (μmol m^−2^ s^−1^) measured with Vaisala GMP252 and COZIR at one *P. avium* L. tree in Jena, Germany, in September 2019 (1-week field data pooled; shape depicts data from three chamber devices). Pearson’s correlation of the relationship was tested. The black solid line shows the trend line and the dashed line is the 1:1 line.

We found a significant correlation between simultaneous measurements of COZIR and Vaisala GMP252 (Pearson correlation coefficient, *R* = 0.95; *P* < 0.001; Figure 4). However, efflux rates measured with the COZIR sensor were consistently lower than rates measured with the Vaisala (up to 11%).

**Figure 5.**
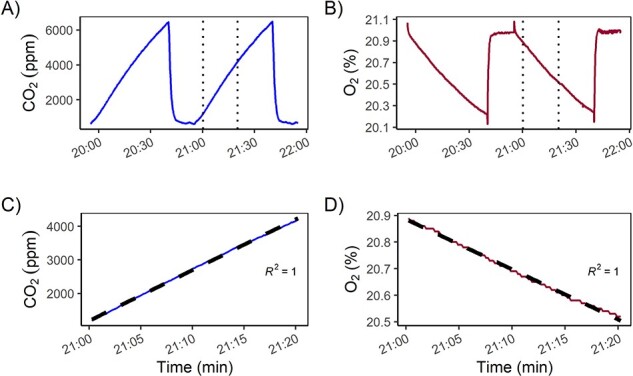
Raw data output of one chamber device installed at one *P. avium* L. tree in Jena, Germany, in September 2019. (A) Increase of CO_2_ and (B) decrease of O_2_ is shown for two consecutive measurement cycles (each cycle: 45 min). Sharp changes in concentration at the end of each cycle reflect flushing the system with ambient air. Dashed lines show 20-min time interval for flux calculation; (C) The 20-min time interval of CO_2_ increases with linear fit (dashed line) and (D) 20-min time interval of O_2_ decrease with linear fit (dashed line). The flushing period and the following 5 min were discarded, before the linear fit was applied.

### CO_2_ and O_2_ flux measurements in the field

During a typical measurement cycle (Figure 5A and B), CO_2_ rapidly increases from atmospheric levels (~400 p.p.m.) to ~6000 p.p.m. (depending on season and time of day) within a 45-min period while the net O_2_ decrease is ~0.7%. Following a typical cycle, it takes ~15-min flushing period (starting at 20:40h in Figure 5) to reach initial concentrations again. For analysis, we focus on the initial 20 min of measurement (beginning 5 min after pumping stopped), for which we assume linearity. In our example, over the 20-min period, we observed changes in the O_2_ and CO_2_ concentration of 4000 p.p.m. and 0.4%, respectively (Figure 5C and D). Relative humidity can vary up to ~8% over the 20-min time interval (change of 1560 p.p.m. at standard condition of 20°C, 945 hPa).

**Figure 6.**
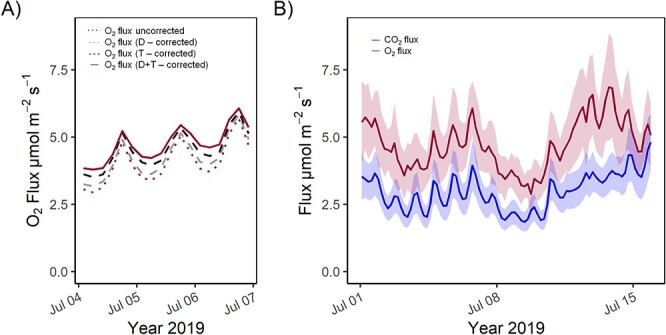
Field data of three mature poplar trees with (A) calculated O_2_ fluxes (4-h mean) according to Eq. (5) over 3 days in July 2019 (Thuringia, Germany, *n* = 3). Uncorrected data and corrected data for O_2_ are shown. After the correction for dilution effect (correction 1; see Eq. 1), temperature correction (correction 2; see Eq. 7) was applied. (B) Calculated CO_2_ (blue) and corrected O_2_ fluxes (red) according to Eq. (5) over 14 days in July 2019 (Thuringia, Germany, *n* = 3 ± SD).

Calculated fluxes (4 h mean) from the field application test on three poplar trees are presented in Figure 6. Two correction steps for O_2_ fluxes were implemented: correction (i) dilution effect on O_2_ (CO_2_ and H_2_O) and correction (ii) temperature effect on LuminOx readings. Dilution correction (including self-dilution) results in an increase of the mean daily fluxes by 5.6 ± 2.2% (Figure 6A), and the subsequent temperature correction from LuminOx readings as determined by our laboratory test results in an increase of the fluxes (daily mean increase of 12.7 ± 4.5%; Figure 6A). Over the 2-week measurement period, CO_2_ efflux is lower than O_2_ influx (Figure 6B); this would result in daily mean ARQ of 0.63 ± 0.06.

## Discussion

In our study, we were able to show that the combination of three low-cost sensors (CO_2_, O_2_, H_2_O) allows reliable and quasi-continuous measurements of CO_2_ and O_2_ stem gas exchange under field conditions. Being affordable, highly mobile and independent of additional infrastructure like local power supply or external logging devices makes our setup highly attractive for application in remote ecosystems or for measuring many individuals and/or widely dispersed trees simultaneously.

### Technical aspects and sensor performance

The sensors installed in our chamber design produced robust measurements. Obviously, such low-cost devices have caveats that one has to be aware of. Our chambers followed a non-steady state incubation design, aiming to measure concentration changes of several thousand p.p.m.; therefore, we only tested the sensors’ performance of measuring relatively big changes of concentration. Especially for the O_2_ sensors, measurements of known concentrations revealed high variability between sensors and—for individual sensors—a significant offset between measured and known concentrations, making accurate measurements of absolute concentrations questionable. Accuracy for O_2_ measurements in terms of absolute values is 20 times lower than for CO_2_ (see sensor specifications in Table 1).

For the COZIR sensors, one important limitation was the effect of sensor drift over time (Table 2). According to our findings, the sensors can be operated without loss of precision for a maximum of 18 days before a new calibration is required. Since calibration has to be done in the laboratory, it is recommended to keep additional calibrated sensors in stock for rapid exchange in the field. Especially when planning to operate a high number of chambers simultaneously, one should consider manufacturing a multi-sensor calibration unit similar to ours for efficient re-calibration.

Direct comparison of the COZIR sensor to the more expensive Vaisala GMP252 showed an offset between the two sensor types, with the COZIR measuring on average 11% smaller fluxes than the Vaisala (Figure 4). Since this comparison was performed under field conditions with unknown concentrations, it is impossible for us to conclude which of the sensor types has the better accuracy. We did not test or re-calibrate the Vaisala in the lab, instead we relied on the manufacturer calibration. The COZIR sensors, on the other hand, were calibrated and thoroughly tested under various laboratory conditions, but calibration was done in dry air, so it is possible that humidity affected the measurements under field conditions. However, the respiration measurements of germinating wheat seeds matched expectations (see below), which indicates that the COZIR works reliably under a realistic humidity range of typical field measurements (50–85%). Assuming that the differences between the Vaisala and the COZIR sensor were due to measurement errors of the COZIR sensor, we would have to consider correction of the field test by up to 11%, thus reducing the observed imbalance between measured O_2_ and CO_2_ fluxes. We would like to point out, however, that this offset is smaller than the observed differences in the field application test.

Field tests in a tropical rainforest (Tanguro ranch, Matogroso, Brazil) indicated complete sensor failure at relative humidity levels between 95 and 100%. One possible solution for tropical applications of stem chambers can be to use humidity traps as shown by Brecheisen et al. 2019 for a field-portable soil gas analyzer. Brändle and Kunert (2019) presented a stem chamber design with an implemented low-cost CO_2_ sensor type MH-Z14A (Winsen Electronics Technology Co., Ltd, Zhengzhou, China). They found good agreement of their device with a portable infrared gas analyzer (Li-8,100; Li-COR Inc., Lincoln, NE, USA) under tropical rainforest conditions at high temperature and high humidity. However, their chamber design was still limited to CO_2_ measurements.

We found two problems that were specific to the LuminOx sensors. First, changes in temperature did affect LuminOx sensor reading. At lower temperatures (5–15°C), O_2_ fluxes based on sensor readings significantly underestimated actual O_2_ fluxes and required a temperature correction (Eq. (7)). Our data indicated that the effect of temperature on sensor readings can differ between individual sensors. In our approach, we used the average correction determined by measuring 10 individual sensors, but correction parameters for individual sensors deviated from the mean by as much as ±8% (see S7 available as Supplementary data at *Tree Physiology* Online). In order to avoid this deviation, correction functions for each sensor individually can be applied. We only tested sensor performance between 5 and 25°C, so it remains uncertain how the sensors behave at temperatures outside of this temperature range. Second, the fact that the sensor does not support adjustment of the manufacturer calibration parameters makes working with this sensor less convenient. However, since we observed no critical sensor drift as with the COZIR sensor, this issue was less of a problem, but it should still be kept in mind when considering using this sensor. In addition to the abovementioned temperature correction, the O_2_ sensors also require a dilution correction to compensate for apparent changes in O_2_ concentration resulting from concentration changes of other gases (mainly CO_2_ and H_2_O). Please note that this is not a correction resulting from technical issues of the sensors, but is a general requirement when measuring concentrations of non-trace gases like oxygen. In the dilution correction, O_2_ self-dilution outweighed the dilution by other gases (CO_2_ and H_2_O), resulting in an increase of ~ 6% (two-week average) for calculated O_2_ fluxes after correction. In our application, we used the relative humidity sensor SHT-85 successfully as this sensor responded quickly (within seconds) to changes in relative humidity, whereas the integrated COZIR RH sensor often underestimated actual humidity levels in the chambers as their response time is very slow to the increasing humidity (see S1 and S2 available as Supplementary data at *Tree Physiology* Online).

To evaluate actual sensor performance under realistic conditions, we measured respiration of germinating wheat seeds. This test allowed testing the sensors over a wider range of concentration changes and within a realistic humidity range. Wheat seeds are a suitable biological model system for this purpose as their carbohydrate-based respiration during the initial germination implies equal CO_2_ and O_2_ fluxes. Results from these wheat seed measurements confirmed that the sensors can reproduce expected values and work under field-humidity levels (Figure 3). The test also underlined the shortcomings of the low-cost sensors with respect to absolute concentration measurements: while the sensors showed similar concentration changes (slopes) during the incubation, absolute concentration measurements at any given time were subject to major offset biases, especially for the O_2_ sensors (see also Figure 2).

To ensure gas tightness, we decided to seal our chambers by means of closed-porous cell foam. The area of the tree stem covered by foam was relatively large compared to the chamber headspace area. Gas exchange for any live tissue underneath the area covered by the foam has to occur via an alternative surface, and some of it will occur via the chamber headspace surface. As a rule of thumb, one may assume that roughly half of the area covered by the foam should be considered as effectively being part of the chamber area. In any case, assuming that the resulting effect is identical for CO_2_ and O_2_, we postulate that the area covered by foam has no impact on the gas exchange ratio or the ARQ.

### Beyond CO_2_: potential application of simultaneous CO_2_ and O_2_ flux measurements in ecosystem and ecophysiological research

The combination of simultaneous CO_2_ and O_2_ measurements in a one-chamber design allows new additional research questions to be addressed. It could be used for the detection of respiratory substrate shifts during stress by calculating the ratio of CO_2_ efflux to O_2_ influx like demonstrated by Fischer et al. (2015) in a greenhouse experiment using Raman spectroscopy. Embedded in the correct experimental design, it could also help to quantify actual rates of local *in situ* respiration by disentangling respiratory CO_2_ production and O_2_ consumption from the effect of other post-respiratory processes (see Hilman et al. 2019 for a detailed discussion). Using simultaneous measurements of CO_2_ and O_2_ fluxes in multiple tree species, they observed a significant mismatch in the amount of CO_2_ emitted vs the amount of O_2_ consumed, which they interpreted as the effect of a variety of whole-tree processes on locally measured CO_2_ concentrations, like non-photosynthetic refixation or stem xylem transport of CO_2_ away from (e.g., to canopy) and to (e.g., from roots) the site of measurement. Data from our initial field test also indicate mismatches between CO_2_ and O_2_ fluxes (Figure 6B), which could be further explored in future experiments. Experimental approaches may include, for example, simultaneous CO_2_ and O_2_ measurements at different stem heights and in the canopy to quantify the effect of vertical gas transport. Combining flux measurements with ^13^C isotope labeling of stem tissue in the dark could help to quantify the postulated non-photosynthetic CO_2_ uptake in tree stems due to PEPC activity. With slight modifications, our chamber design may also be useful for measuring other ecosystem components like soil, root, branch or leaf fluxes.

Furthermore, data provided by our device can serve as important input and calibration variables for mechanistic models of tree and stem functioning. For instance, Salomón et al. (2020) developed TreSpire, a process-based model, which couples carbon and water fluxes at the organ (stem) level. Implementation of combined CO_2_ and O_2_ data can provide crucial information to constrain the model parameter space. In this way, key parameters used to estimate overall tree respiration at large spatial scales—growth respiration coefficient, respiration sensitivity to temperature (Q_10_) and basal maintenance respiration (Atkin et al. 2017)—could be accurately estimated. Such insights are much needed to improve model predictions and advance our understanding on stem respiration, for which currently measurements of CO_2_ efflux at breast height are commonly used as estimates for whole-tree respiration, even though stem CO_2_ efflux does not reflect respiration rates of underlying tissues (e.g., Teskey et al. 2008, Trumbore et al. 2013, Darenova et al. 2018, Salomón et al. 2020).

## Conclusion

We present a versatile low-cost chamber setup for measuring CO_2_ and O_2_ fluxes between tree stems and the atmosphere. Adaptation of the general setup to other applications (e.g., soil or branch measurements) should technically be relatively easy. We showed that low-cost sensors are prone to drift over time and/or require temperature correction. Our study also points out that O_2_ sensors require dilution correction to get accurate O_2_ data that are not biased from concentration changes of other gases (CO_2_ and H_2_O). Using both CO_2_ and O_2_ measurements in the correct experimental design provides additional information on tree physical and physiological processes like xylem CO_2_ transport, post-respiratory enzymatic fixation of CO_2_ and subcortical photosynthetic uptake of respired CO_2_.

## Supplementary Material

SupplementaryData_tpab022
