# Supplementary material for: Low-cost chamber design for simultaneous CO2 and O2 flux measurements between tree stems and the atmosphere
Source: Tree Physiol. 2021 Mar 2;41(9):1767–80. doi: 10.1093/treephys/tpab022 (PMC8441941; doi:10.1093/treephys/tpab022)
Supplement: SupplementaryData_tpab022 [file supplementarydata_tpab022.docx]

### **Supplementary data**

#### Low-cost chamber design for simultaneous CO_2_ and O_2_ flux measurements between tree stems and the atmosphere

### **Supplementary data S1 and S2**

We tested two different humidity sensors in the laboratory- the internal relative humidity sensor integrated in the COZIR sensor, and the SHT-85. Both sensors were exposed to air that was pumped through a dew point generator (DPG, LI-610, LI-COR Biosciences, Bad Homburg, DE) to create known levels of humidity. Our tests showed very fast response and accurate measurements for the SHT-85. The internal humidity sensor of the COZIR, however, reacted much slower to changes and consistently underestimated the humidity. In the beginning and during the field tests we used the COZIR data for dilution corrections, but this will underestimate humidity (S2). For the humidity correction, we recommend using the more accurate alternative.


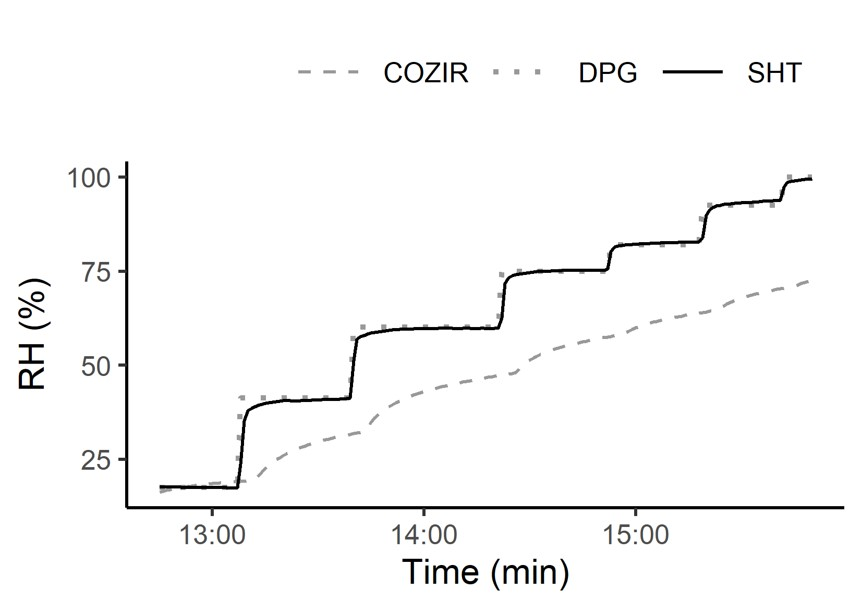


**S1** Relative humidity measurements (%) of the external humidity sensor (SHT-85, n = 2) and COZIR sensors (n = 10) over time within the calibration and testing unit in the laboratory. Different humidity levels were set with a Dew Point Generator (DPG, LI-610, LI-COR Biosciences, Bad Homburg, DE).


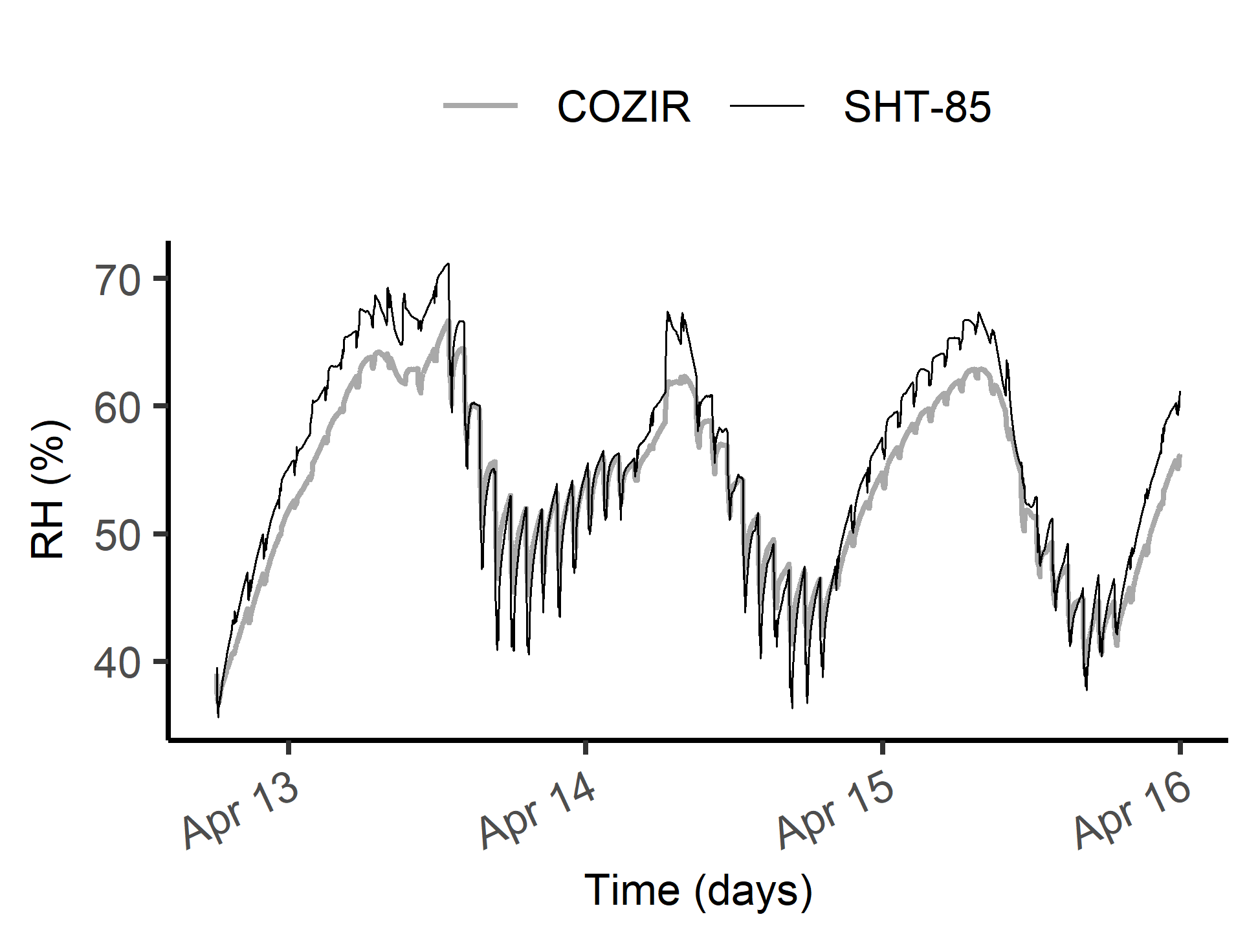


**S2** Relative humidity measurements (%) of the external humidity sensor (SHT-85) and COZIR RH sensor within the headspace of the stem chamber over time (n = 3). Three chambers were installed at three positions on the same tree (*Prunus avium* L., mean stem diameter: 105 cm) and measured relative humidity for three days in April 2020 in Jena, Thuringia, Germany.

### **Supplementary Data S3**

| 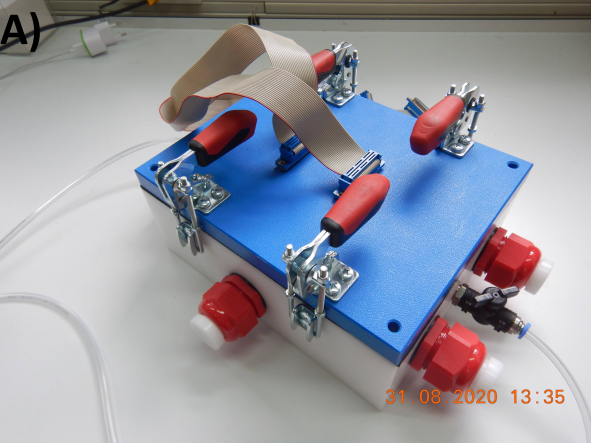 | 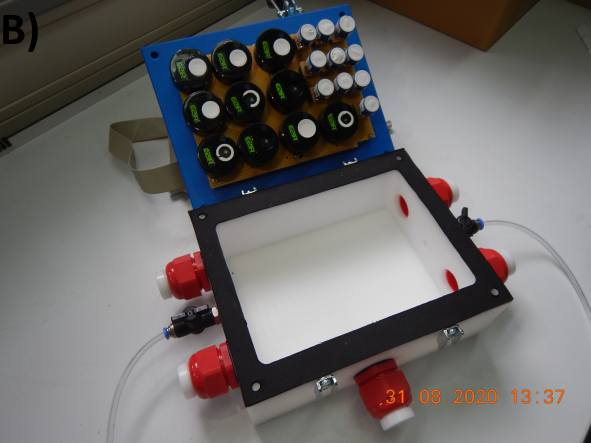 |
| --- | --- |
| 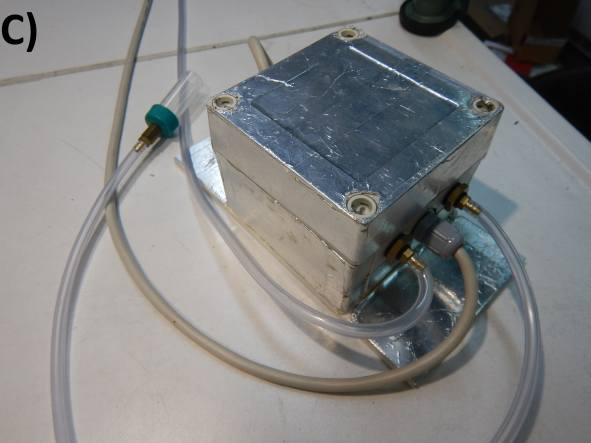 | 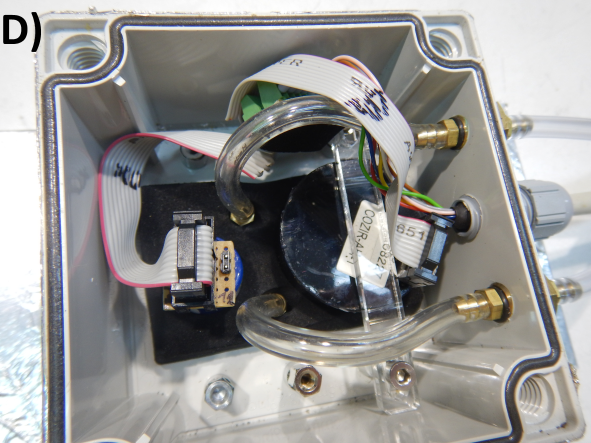 |
| 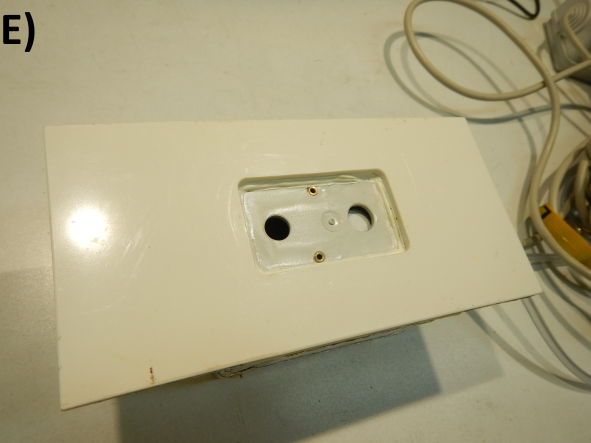 | 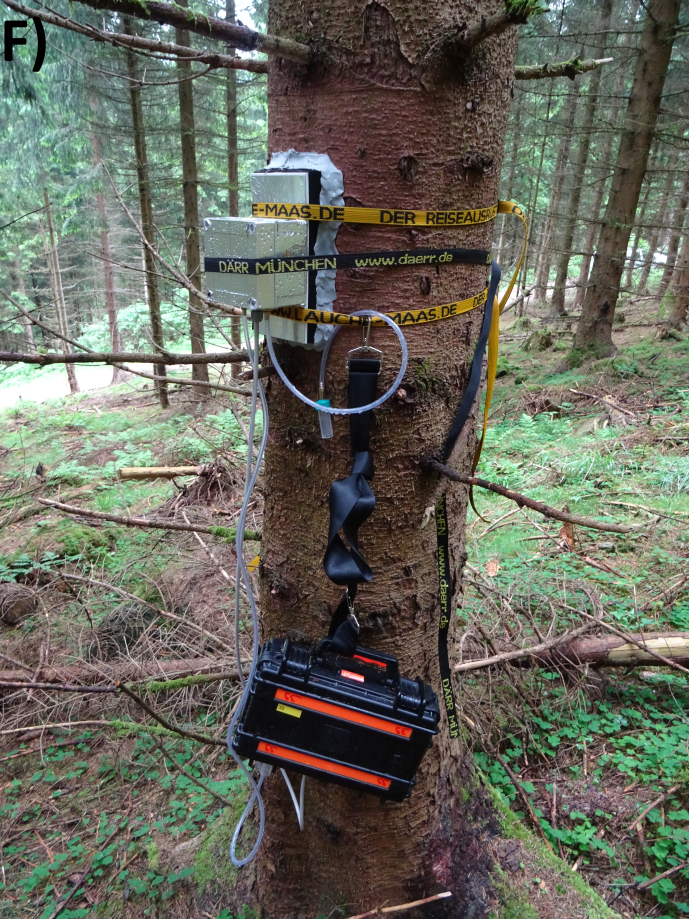 |

**S3** A) and B) Calibration and testing unit for 10 COZIR and 10 LuminOx sensors. C) Sensor box for COZIR and LuminOx sensors covered with aluminium foil, D) COZIR and LuminOx sensors placed inside the waterproof housing. E) Sensor box and base plate of the chamber module and F) Installation in the field with sensor box and waterproof transport-case.

### **Supplementary Data S4**

Details for custom made datalogger: The custom-built data logger comprises an Arduino Mega 2560 R3 with added data logging shield and a 16x2 RGB LCD shield.

The LCD shield displays actual CO_2_ and O_2_ values and other parameters like time/date, file name and accumulation/flushing times, which can be set using the buttons.

The data logging shield holds the SD memory card for data storage (4 MB, more than one year of operation) and the real-time-clock chip for time-stamping the measurement values. The prototyping area of the shield also holds the MOSFET driver for the 6V pump (NMP015S from KNF, ca. 1 L/min due to running at 5V).

The program (a.k.a. „sketch“) on the arduino has the following structure: On powering up, the last known parameters (pump/flush duration, file number, measurement interval) are loaded from the EEPROM. Both sensors are set into poll mode to not flood the arduino in buffer. The SD card is checked for existing data files and a new one is being created to hold this session's data. After this initialisation, the standard main loop is entered, which runs ad infinitum. The main loop checks if pumping is due or not, then checks and resolves any user input on the LCD shield buttons, then polls both sensors for all data they can possibly measure (O_2_: O_2_partial pressure, temperature, barometric pressure, O_2_ percentage value; CO_2_: CO_2_ in ppm, temperature, RH). After all data has been received it’s checked for integrity then formatted and saved to the file on the SD card.

### **Supplementary Data S5**

**S5** Component list of the stem chamber (main parts)

| Component description | Quantity | Supplier | Part number | Unit price [€] |
| --- | --- | --- | --- | --- |
| Transport-case | 1 | Dyntronic-plenty.de | 72601-K | 25.- |
| Fibox Grey ABS Enclosure 100mmx100mmx75mm IP67 | 1 | RS | 498-4025 | 8.- |
| Ratchet straps | 3 | Various |  | 15.- |
| Closed cell EPDM foam mat 40mm | 1 | luxco24.de | 84700200040 | 2.- |
| Arduino Mega | 1 | RS | 7154084 | 35.-. |
| Arduino Display Shield negative | 1 | exp-tech.de | EXP-R15-123 | 30.- |
| Arduino Datalogger Shield | 1 | exp-tech.de | EXP-R15-003 | 1.5.- |
| Generic SD card (4GB) | 1 | Various |  | 10.- |
| Components for circuit board assembly (self-made design) | 1 |  |  | 10.- |
| Battery 50.000mAh* | 1 | Xtpower.de |  | 150.- |
| Membrane pump, NMP015S, 6VDC | 1 | KNF | NMP015B | 70.- |
| CO_2_ Sensor 10k ppm | 1 | GSS Ltd. |  | 90.- |
| O_2_ Sensor 0...25% | 1 | SST Ltd. |  | 50.- |
| H_2_O Sensor SHT-85 | 1 | RS |  | 23.- |
| *We used this power bank to achieve up to 12 days of continuous operation. Smaller power banks (20 Ah) are much cheaper (ca. 30 €) and can be more easily acquired, but only allow ca. 4-5 days of continuous operation. |  |  |  |  |
|  |  |  | Total [€]: 519.5 | |

### **Supplementary Data S6**

Correction of measurement data (O_2_) for the dilution effect of changing H_2_O and CO_2_ concentrations; Description of the original approach described by Keeling et al. (1998); extended by a H_2_O correction:

We can convert our measurements of apparent mole fraction to relative changes on the per meg scale while correcting for the diluting effect of changes in the simultaneously measured CO_2_ concentration as follows (using the Kozlova *et al.* (2008) modified version of the Stephens *et al.* (2007) equation):

|  | $\delta(O_{2}/N_{2})=\frac{\delta X_{O_{2}}+S_{O_{2}}\left( \left[ {CO}_{2} \right]_{sample}-\left[ {CO}_{2} \right]_{reference} \right)+S_{O_{2}}\left( \left[ H_{2}O \right]_{sample}-\left[ H_{2}O \right]_{reference} \right)}{S_{O_{2}}(1-S_{O_{2}})}$ |  |
| --- | --- | --- |

where δ(O_2_/N_2_) is the change of the O_2_/N_2_ ratio in per meg, δXO_2_ is apparent mole fraction change. i.e. the differences between the measured apparent mole fraction and the arbitrarily defined reference mole fraction (SO_2_) of 0.20946, multiplied by 10^^6^ to express as ‘per meg’. [CO_2_]_sample_ is the measured CO_2_ concentration in µmol mol-1, [CO_2_]_reference_ is the CO_2_ concentration of the reference cylinders that define the zero point on the Scripps O_2_ scale (363.29 µmol mol^-1^). [H_2_O]_sample_ is given in ppm (conversion from % to ppm, see section 2.5). [H_2_O]_reference_ is 0. The addition of 1 µmol of O_2_ to 1 µmol of dry air results in a change of 4.77 per meg (Keeling et al., 1998), so for comparison of O_2_ and CO_2_ fluxes we divided the results by this factor to get O_2_ concentrations relative to the reference in ppm equivalents, which we used as the basis for all subsequent O_2_ flux calculations.

New mathematical approach (explanation see section 2.5):

$$\delta O_{2,corr}[ppm]=\frac{\delta O_{2,app}[ppm]+{{\delta{CO}_{2}[ppm]\times X}_{O_{2}}}_{t=0}{{+\delta H_{2}O[ppm]\times X}_{O_{2}}}_{t=0}}{1-{X_{O_{2}}}_{t=0}}$$

No significant difference between the two approaches was found for our measurement campaign on three poplar trees (t= 0.0283, p = 0.9776).

General note: The implemented correction function we use within this manuscript for dilution by O_2_, CO_2_ and H_2_O is based on some assumptions that might not always be justified (personal communication Dr. Jelka Braden-Behrens). Among other assumptions, such as constant nitrogen and argon content and a constant O_2_ mole fraction relative to CO_2_ (and H_2_O)-free air, this correction function neglects some second order (mixed) terms (personal communication Dr. Jelka Braden-Behrens). These are basically the same assumptions as those that underlie the often-used CO_2_ and O_2_ dilution correction (see eg. Kozlova *et al.,* 2008).

Keeling RF, Manning AC, McEvoy EM, Shertz SR. 1998. Methods for measuring changes in atmospheric O2 concentration and their application in southern hemisphere air. *Journal of Geophysical Research: Atmospheres* 103(D3): 3381-3397.

Kozlova EA, Manning AC, Kisilyakhov Y, Seifert T, Heimann M. 2008. Seasonal, synoptic, and diurnal‐scale variability of biogeochemical trace gases and O2 from a 300‐m tall tower in central Siberia. *Global Biogeochemical Cycles* 22(4).

Stephens BB, Bakwin PS, Tans PP, Teclaw RM, Baumann DD. 2007. Application of a differential fuel-cell analyzer for measuring atmospheric oxygen variations. *Journal of atmospheric and oceanic technology* 24(1): 82-94.

### **Supplementary Data S7**


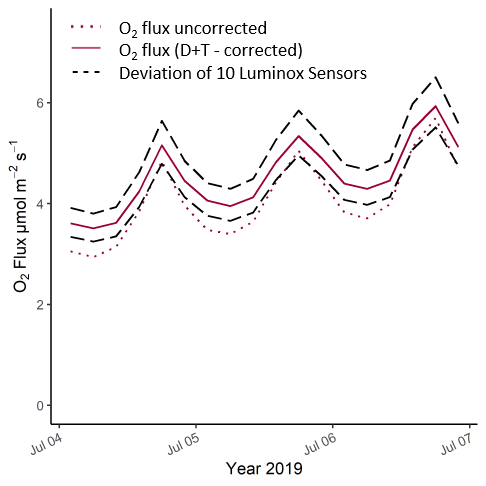


**S7** Calculated O_2_ fluxes (4h mean) according to eq. [6] over 3 days in July 2019 (Thuringia, Germany, n = 3). Uncorrected data (dotted line) and corrected data (dilution (D-) and temperature (T-) corrected, solid line) for O_2_ are shown. Black dashed line represents the extremes of 10 sensors, applying the formulas *SC(*$O_{2})$ = $-0.010 x T \left( in ^{\circ}C \right)+1.40$ and *SC(*$O_{2})$ = $-0.009 x T \left( in ^{\circ}C \right)+1.20$.
